# Influence of socioeconomic status on oral disease burden: a population-based study

**DOI:** 10.1186/s12903-021-01970-w

**Published:** 2021-11-30

**Authors:** Edson Hilan Gomes de Lucena, Rênnis Oliveira da Silva, Maria Letícia Barbosa, Elza Cristina Farias de Araújo, Antonio Carlos Pereira, Yuri Wanderley Cavalcanti

**Affiliations:** 1 Federal University of Paraiba, João Pessoa, Brazil; 2 University of Campinas, Campinas, Brazil

**Keywords:** Oral health, Global Burden of Disease, Socioeconomic factors

## Abstract

**Background:**

Dental caries is associated with Biological, behavioral, socioeconomic, and environmental factors; however, socioeconomic status is a distal determinant of dental caries development that modulates exposure to risk and protective factors. This study aimed to analyze the socioeconomic factors associated with the concentration of oral diseases in a population-based study in Brazil.

**Methods:**

This is a quantitative, analytical, cross-sectional study based on secondary data from the SB São Paulo 2015 epidemiological survey. A total of 17,560 subjects were included. The concentration of oral disease in the population was estimated by the oral disease burden (ODB) variable. The ODB consists of four components: dental caries; tooth loss; need for dental prosthesis and periodontal condition. Thus, the total score on the ODB could vary between 0 and 4, with the highest score indicating the worst possible situation. ODB was analyzed in multivariate negative binomial regression, and multivariate binary logistic regression analysis. The following factors were included as independent variables: age group, skin color, socioeconomic factors, family income and Oral Impact on Daily Performance (OIDP).

**Results:**

In the sample, 86.9% had no minimum ODP component. Negative multivariate binomial regression showed a statistically significant relationship (*p* < 0.005) between ODB and all variables analyzed (skin color, family income, education, OIDP results and age range). The adjusted multivariate binary logistic regression showed that the individuals most likely to have at least one component of ODB were nonwhite (25.5%), had a family income of up to R$ 1500.00/month (19.6%), had only completed primary education (19.1%), and reported that their oral health had an impact on their daily activities (57.6%). Older adults individuals were two times more likely than adolescents to have an ODB component.

**Conclusions:**

ODB is associated with factors related to social inequality. Adults and older adults individuals had the highest cumulative number of ODB components.

## Background

Oral diseases are a major global public health problem with a high prevalence and large negative impacts on individuals, communities, and society. These diseases disproportionately affect the poorest and most marginalized groups in society and are closely linked to socioeconomic status and social determinants of health [1].

Biological, behavioral, socioeconomic, and environmental factors are associated with dental caries and its consequences [2]. Socioeconomic status is a distal determinant of dental caries development that modulates exposure to risk and protective factors as well as access to oral health services [3]. These inequalities in the distribution of dental caries have been reported in different countries [4, 5]. In this context, the World Health Organization (WHO) determined that studies on socioeconomic inequalities in the distribution of oral diseases and on the design of strategies to increase access should be research priorities for the twenty-first century [6].

To provide greater equity in dental care for socially disadvantaged groups, it is necessary to understand populations’ specific characteristics, their socioeconomic status, and, above all, the influence of these factors on their health-related behavior. Such understanding will contribute to reducing disparities in oral health [7, 8]. There is an urgent need for appropriate actions and services to effectively address disparities in the oral health of disadvantaged groups [9].

The use of information on the living conditions of the population is essential given the need to ensure that the provision of health care is guided by equity. They should underpin analyses of the health-disease status of the population in each territory and should inform the planning and development of actions aimed at those who need them the most. That is, it must be used to inform the action, and the actions must be equitable [10].

For the implementation of a health surveillance model, as expected by the Brazilian Unified Health System (*Sistema Único de Saúde*, SUS), accurate epidemiological information is necessary because it indicates profiles and trends in health conditions [11]. Based on this premise, it is expected that dentists working in primary health care will have the information and knowledge about the population in the territory under their care that is necessary to strengthen and direct their actions towards those who most need them [12].

With the objective of eliminating inequalities caused by adverse social conditions, the SUS advocates equity [13]. Universal health care systems offer an opportunity for dental health services to become more integrated into the broader health care system and to be more accessible and meet the oral health needs of the population [14].

Socioeconomic status is historically associated with inequalities in oral health [1, 15–17]. However, socioeconomic factors related to the prevalence and severity of oral diseases in portions of the population still need to be elucidated. Identifying which factors are associates in the concentration of diseases within the population can help health care managers and health professionals to intervene more efficiently and equitably. To this end, this study aimed to analyze the socioeconomic factors associated with the concentration of oral diseases in a population-based study in Brazil.

## Methods

This is a quantitative, analytical, cross-sectional study based on secondary data from the SB São Paulo 2015 (SBSP-2015) epidemiological survey. The data used in this study were extracted from the public dataset of the study, which is available online [18].

The SBSP-2015 study was approved by the Human Research Ethics Committee of the School of Dentistry of Piracicaba (FOP-UNICAMP) and registered under number 1,211,025; CAEE no. 46788215.9.0000.5418. Everyone who participated in the study signed an informed consent form.

The SBSP-2015 was a population-based study that aimed to evaluate population-based oral health and socioeconomic status in different age groups in the state of São Paulo, Brazil. The complex sample was divided into the six domains of the state (including the capital, the metropolitan region of São Paulo and 15 Regional Health Districts). A two-stage selection process with a selection probability proportional to the population size was used in the sampling design: (1) 178 cities, including the capital São Paulo, were designated primary stage units (PSAs), and (2) two census tracts were randomly selected in each selected city (secondary stage units, SSUs, totaling 390 areas), including 36 areas within São Paulo state. Data relative to the age groups of 15 to 19 years (n = 5585), 35 to 44 years (n = 6051) and 65 to 74 years (n = 5951) were used, and a total of 17,560 subjects were included.

Examiner training procedures, data collection methods and the variables included in the study were previously described in other studies [19–24]. The concentration of oral disease in the population was estimated by the oral disease burden (ODB) variable, which was the outcome of this study. The ODB variable consists of four components: dental caries; tooth loss; need for dental prosthesis and periodontal condition (gingival bleeding, tartar and periodontal pocketing), which are categorized as "0" (absence of the condition) or "1" (presence of the condition).

As the ODB indicator is composed of four components, the total score on the ODB could vary between 0 (absence of the assessed condition) and 4 (the presence of all assessed conditions). Thus, the highest score indicating the worst possible situation since it indicated that the subject presented all the evaluated conditions.

ODB was analyzed in two ways. The first was multivariate negative binomial regression, in which it was categorized according to five levels: 0 indicated the absence of all components of ODB, 1 indicated the presence of one of the components, 2 indicated the presence of two components, 3 indicated the presence of three components, and 4 indicated the presence of all components of ODB. The second method was multivariate binary logistic regression analysis, in which the variable was dichotomized as "no ODB" (the absence of the evaluated indicators) and "ODB" (the presence of at least one evaluated indicator).

The following demographic data were included as independent variables: age group, subdivided into "adolescents" (15 to 19 years old), "adults" (35 to 44 years old), and "older adults" (65–74 years old); skin color, dichotomized as “white” and “non-white"; and socioeconomic data, including education, which was dichotomized as “primary education” and “secondary or higher education”, and family income, which was dichotomized as "up to R$1500.00/month" and "over R$1500.00/month". The Oral Impact on Daily Performance (OIDP) scale score was dichotomized as "impact" for people who answered "yes" to one or more questions and "no impact" for participants who answered "no" to all 9 questions on the questionnaire, which was used to assess quality of life through the impact of oral health on daily living [25, 26].

Analyses were performed using the *Statistical Package for Social Sciences* (IBM-SPSS, v.24, IBM, Chicago, IL) software considering a 95% confidence interval and a statistical significance of 5%. ODB fit a negative binomial distribution, and a negative binomial multiple regression analysis was performed. All independent variables were included in the negative binomial multiple regression model. To adjust the model, variables with *p*-values > 0.20 were removed. From the coefficients of the negative binomial regression model, the effect magnitudes were estimated by using prevalence ratios (PR) and 95% confidence intervals.

After the adjusted negative binomial regression model was obtained, the dependent variable (ODB) was dichotomized and analyzed according to a binary logistic regression model to determine the effect of the independent variables included in the adjusted model on the chance of an individual having ODB ≥ 1. For this purpose, odds ratios (OR) and 95% confidence intervals were estimated. Then, multiple correspondence analysis (MCA) was performed to determine the interaction/proximity of each independent variable category with the possible outcomes of the dependent variable (the presence and absence of ODB) [27]. The MCA resulted in a contingency diagram that enabled a qualitative analysis of the effect of the interaction between the independent and dependent variables to complement the multivariate logistic regression.

## Results

ODB was present in 86.9% of the sample (n = 17,560), which consisted of 31.7% adolescents, 34.5% adults, and 33.9% older adults individuals. Of these, 63.6% were self-reported as non-white, 45.5% had a family income greater than R$1501.00/month, 54.4% had completed primary education, and 56.6% of the sample indicated that their oral health had an impact on their daily activities, as evaluated by the OIDP (Table 1).

**Table 1:** Descriptive analysis of the participants according to their profiles

Variables	N (%)
*Skin color*	
Non-white	6386 (36.4)
White	11,174 (63.6)
*Family income*	
Up to R$1500.00/month	7030 (40.0)
Over R$1501.00/month	7988 (45.5)
No information	2542 (14.5)
*Education*	
Primary education	9555 (54.4)
Secondary or higher education	6720 (38.3)
No information	1285 (7.3)
*OIDP*	
Impact of oral health on daily activities	6829 (38.9)
No impact of oral health on daily activities	9947 (56.6)
No information	784 (4.5)
*Age group*	
Teenager (15 to 19 years old)	5558 (31.7)
Adult (35 to 44 years old)	6051 (34.5)
Older adults (65 to 74 year old)	5951 (33.9)
*ODB*	
No ODB	2280 (13.0)
ODB	15,260 (86.9)
No information	20 (0.1)
Total	17,560 (100.0)

Negative multivariate binomial regression showed a statistically significant relationship (*p* < 0.005) between ODB and all variables analyzed (skin color, family income, education, OIDP results and age range) (Table 2). The highest prevalence of ODB components was observed in non-white individuals are 1.08 times more likely to increase a score on the ODB indicator (PR = 1.084; IC95%: 1.058–1.111), even as with a family income of up to R$1500.00/month are 1.08 times more likely (PR = 1.078; IC95%: 1.054–1.103), those who had only completed primary education are 1.16 times more likely (PR = 1.161; IC95%: 1.126–1.196), and those who indicated that their oral health had an impact on their daily activities are 1.25 times more likely (PR = 1.258; IC95%: 1.229–1.288). Older adults individuals (65 to 74 years old) are 2.85 times more likely (PR = 2.851; IC95%: 2.716–2.992) and adults (35 to 44 years old) are 2.71 times more likely (PR = 2.712; IC95%: 2.590–2.841) were more likely than adolescents (15 to 19 years old) to have an additional ODB component (Table 2).

**Table 2:** Multivariate and adjusted negative binomial regression of ODB and the independent variables

	B	*p*-value	PR	95% confidence interval	
				Lower	Upper
*Skin color*					
White	Ref		1		
Non-white	0.081	< 0.001	1.084	1.058	1.111
*Family income*					
Up to R$1500.00/month	0.075	< 0.001	1.078	1.054	1.103
Over R$1501.00/month	Ref		1		
*Education*					
Primary education	0.149	< 0.001	1.161	1.126	1.196
Secondary or higher education	Ref		1		
*OIDP*					
No impact of oral health on daily activities	Ref		1		
Impact of oral health on daily activities	0.230	< 0.001	1.258	1.229	1.288
*Age group*					
Teenager (15 to 19 years old)	Ref		1		
Adult (35 to 44 years old)	0.998	< 0.001	2.712	2.590	2.841
Older adults (65 to 74 year old)	1.048	< 0.001	2.851	2.716	2.992

B: Regression coefficient; p-value: Statistical significance; PR: Prevalence ratio

The adjusted multivariate binary logistic regression showed that the individuals most likely to have at least one component of ODB were non-white are 25% more chance (OR = 1.255; IC95%: 1.180–1.335), had a family income of up to R$1500.00/month are approximately 20% more chance (OR = 1.196; IC95%: 1.126–1.271), had only completed primary education are 19% more chance (OR = 1.191; IC95%: 1.120–1.266), and reported that their oral health had an impact on their daily activities are 57% more chance (OR = 1.576; IC95%: 1.479–1.679). Older adults individuals (OR = 14.807; IC95%: 10.241–21.409) were fourteen times more likely than teenager to have an ODB component (Table 3).

**Table 3:** Multivariate and adjusted binary logistic regression of the ODB indicator and the independent variables

	B	*p*-value	OR	95% confidence interval	
				Lower	Lower
*Skin color*					
White	Ref		1		
Non-white	0.227	< 0.001	1.255	1.180	1.335
*Family income*					
Up to R$1500.00/month	0.179	< 0.001	1.196	1.126	1.271
Over R$1501.00/month	Ref		1		
*Education*					
Primary education	0.175	< 0.001	1.191	1.120	1.266
Secondary or higher education	Ref		1		
*OIDP*					
No impact of oral health on daily activities	Ref		1		
Impact of oral health on daily activities	0.455	< 0.001	1.576	1.479	1.679
*Age group*					
Teenager (15 to 19 years old)	Ref		1		
Adult (35 to 44 years old)	− 0.115	0.270	0.892	0.727	1.093
Older adults (65 to 74 year old)	2.695	< 0.001	14.807	10.241	21.409

B: Regression coefficient; *p*-value: Statistical significance; OR: Odds ratio

**Fig. 1:**
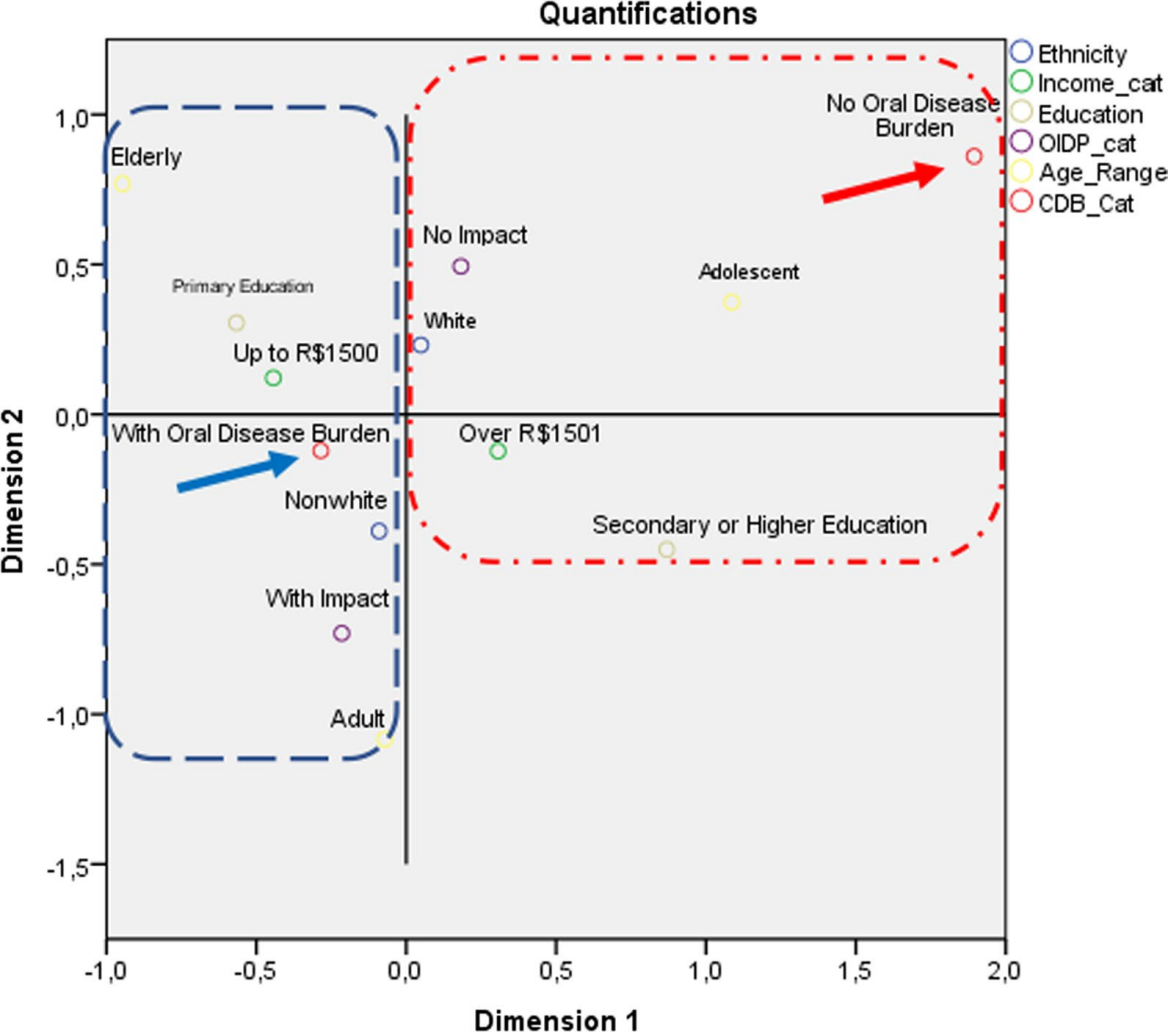
Multiple Correspondence Analysis (MCA) diagram of variables (ethnicity, family income, education, OIDP and age) associated with Oral Diseade Burden (blue circle) and no Oral Diseade Burden (red circle)

Multiple correspondence analysis was performed with all the independent variables that were statistically significant in the multivariate binary logistic regression analysis. Figure 1 shows a greater relationship/proximity between “No oral health burden” and the characteristics “teenager”, “white”, “no impact of oral health on daily activities”, “family income over R$1500.00/month" and "secondary or higher education".

## Discussion

The results of this study reinforce the association between socioeconomic inequalities and the concentration of oral diseases. In addition, it highlights the need to examine access to public oral health services. The distribution of oral diseases occurs heterogeneously in different social groups. The distribution of oral disease in the population is unequal, and it is considered an inequity in health since this unequal can be avoided, and the fact that it persists is unjust [28].

A greater presence of components of ODB (dental caries, tooth loss, the need for dental prostheses, and periodontal conditions) was identified in non-white individuals, those with a low family income, those with few years of study, and those who indicated that their oral health had an impact on their daily activities. This supports the findings of the study, who argue that strong socioeconomic inequalities in oral health mean that poor and vulnerable groups in society are particularly affected [14].

It is relevant to investigate whether the majority population group in Brazil (the brown and black population) is receiving adequate care to reduce the burden of oral diseases [29]. This group is more vulnerable because it has lower levels of education and income [30], poorer overall health outcomes [31] and poorer oral health [32]. However, although they are at higher risk, they are less likely to use the dental health services available [33] and to visit the dentist for preventative care [29].

The association of higher ODB with socioeconomic factors reinforces the need to overcome the exclusiveness of oral health care approaches and to combine broader policy initiatives to combat oral health inequalities at the structural level, with a focus on social issues, determinants of health and shared risk factors between oral diseases and other chronic noncommunicable diseases [34].

The few studies of this higher disease burden demonstrate the need for inclusive educational policies. Cities with better educational policies showed a lower prevalence of untreated dental caries and tooth loss than cities with worse educational policies [35]. Education can also act indirectly on income: the higher an individual’s education level is, the greater his or her possibility of finding a better paid job, which would increase his or her ability to pay for private dental care, among other needs [29]. In addition, the positive impact can manifest as increased knowledge and the adoption of healthy habits [36].

The lower income group had higher percentages of untreated dental caries in all municipalities, regardless of the availability of public policies (sanitation, dental care and education) and the fluoridation of public water supply. The income indicator establishes a nexus with health levels to the extent that it enables individuals to acquire goods and services that promote or rehabilitate health [36].

The adjusted multivariate binary logistic regression model showed that older adults individuals have a fourteen times greater chance than adolescents of having a component of ODB. This demonstrates that the most impacting oral diseases and disorders are cumulative and chronic [37] is that socioeconomic status cumulatively affects oral health throughout life and highlights the importance of this status as an indirect factor in oral health later in life [38].

In this study, we considered different age groups because it is necessary to expand oral health studies beyond children and adolescents to include adults and older adults individuals due to changes in the aging of the population, the increase in life expectancy, and the displacement of the disease burden in the direction of chronic diseases. For this reason, studies on inequalities in the distribution of dental caries among these groups are necessary [3].

The OIDP results were associated with a higher ODB. The analysis of this indicator is relevant because it enables the assessment of oral health-related quality of life (HRQOL). Oral HRQOL is a multidimensional indicator that assesses the extent to which oral diseases affect the daily functioning and the social, emotional and psychological well-being of individuals [39]. The findings corroborate those of other studies that associate the worst individual social conditions with oral health problems and low HRQOL [40–42].

Considering the high concentration of goods and wealth in Brazil and the existence of a health system that includes equity as one of its principles, it is very important for health research and planning to have a systematic understanding of studies that have investigated social inequalities in the prevalence of dental caries [3].

The use of zone and population information in the planning and programming of health services is a major challenge given the initial limitation of professional training and the efforts required by the health surveillance-based model of care, which is based on the premise that information on determinants, risk and protective factors, and damage to health can be monitored to identify vulnerable groups and populations or those with potential for a healthy life [43].

There are compelling reasons to be concerned with resolving health inequalities. The persistence of differences in health based on race/ethnicity or other social factors (such as education) raises moral concerns and upsets the basic notion of justice and human rights.

The current study has some limitations and strengths. In general, this population-based study from the state of São Paulo provides some evidence of the social and economic factors associated with a greater ODB. Although it is not possible to replicate the results for the entire country of Brazil, it is noteworthy that São Paulo is the most populous state in the country, comprising approximately 22% of the Brazilian population [44].

It should be noted that the multiple correspondence analysis should be interpreted as complementary to the logistic regression model because it illustrates the relationships of each category of independent variable with the binary categories of the dependent variable.

Due to the cross-sectional nature of the study, temporal relationships cannot be elucidated. However, the inverse cause may be unlikely given that the components of ODB have low latency in the population, presumably because the contextual characteristics that were evaluated, such as race/color and years of study, were present before the ODB emerged.

The findings of this study may help researchers, oral health professionals and managers in planning and programming oral health services in the SUS. Other studies that analyze the association between oral health diseases and socioeconomic factors, the work of oral health teams, and the organization of the Oral Health Network are necessary to construct an inclusive and effective practice; therefore, it is necessary to approach the people who need oral health services and try to understand their living conditions.

## Conclusion

ODB is associated with factors related to social inequality. In the state of São Paulo, higher ODB was present in those who had only completed primary school, are non-white, those with a low family income, and those whose oral health had some impact on their daily activities. Adults and older adults individuals had the highest cumulative number of ODB components.

## Data Availability

The datasets analysed during the current study are available in the SBSP 2015, https://w2.fop.unicamp.br/sbsp2015/.

## Abbreviations

WHO: World Health Organization
SUS: Brazilian Unified Health System (*Sistema Único de Saúde)*
SBSP-2015: Oral Health São Paulo 2015 (*SB São Paulo 2015)*
PSAs: Primary stage units
ODB: Oral Disease Burden
OIDP: Oral impact on daily performance
PR: Prevalece ratios
OR: Odds ratios
MCA: Multiple correspondence analysis
HRQOL: Oral health-related quality of life
